# Towards Poly(vinylidene fluoride-trifluoroethylene-chlorotrifluoroethylene)-Based Soft Actuators: Films and Electrospun Aligned Nanofiber Mats

**DOI:** 10.3390/nano11010172

**Published:** 2021-01-12

**Authors:** Riccardo D’Anniballe, Andrea Zucchelli, Raffaella Carloni

**Affiliations:** 1 Faculty of Science and Engineering, University of Groningen, Nijenborgh 9, 9747 AG Groningen, The Netherlands; r.carloni@rug.nl; 2 Department of Industrial Engineering, Interdepartmental Centre for Industrial Research in Advanced Mechanical Engineering Applications and Materials Technology (CIRI-MAM), University of Bologna, Viale Risorgimento 2, 40136 Bologna, Italy; a.zucchelli@unibo.it

**Keywords:** P(VDF-TrFE-CTFE), electrostriction, nanofibers, soft actuators

## Abstract

In the pursuit of designing a linear soft actuator with a high force-to-weight ratio and a stiffening behavior, this paper analyzes the electrostrictive effect of the poly(vinylidene fluoride-trifluoroethylene-chlorotrifluoroethylene) polymer in the form of film and aligned electrospun nanofiber mat. An experimental setup is realized to evaluate the electrostrictive effect of the specimens disjointly from the Maxwell stress. In particular, an uniaxial load test is designed to evaluate the specimens’ forces produced by their axial contraction (i.e., the electrostrictive effect) when an external electric field is applied, while an uniaxial tensile load test is designed to show the specimens’ stiffening properties. This electro-mechanical analysis demonstrates that both the film and the nanofiber mat are electrostrictive, and that the nanofiber mat exhibits a force-to-weight ratio ∼65% higher than the film and, therefore, a larger electrostrictive effect. Moreover, both the film and the nanofiber mat show a stiffening behavior, which is more evident for the nanofiber mat than the film and is proportional to the weight of the material. This study concludes that, thanks to its electro-mechanical properties, the poly(vinylidene fluoride-trifluoroethylene-chlorotrifluoroethylene), especially in the form of aligned electrospun nanofiber mat, has high potential to be used as electro-active polymer for soft actuators in biomedical and biorobotics applications.

## 1. Introduction

In the last decades, extensive research has been conducted on electro-active polymers due to their inherent ability to convert electrical energy into mechanical energy, namely, to change size or shape (i.e., bending, contraction, or expansion) when stimulated by an external electric field. As a consequence, electro-active polymers are promising soft actuators for a variety of biomedical and biorobotic applications [1].

**Figure 1:**
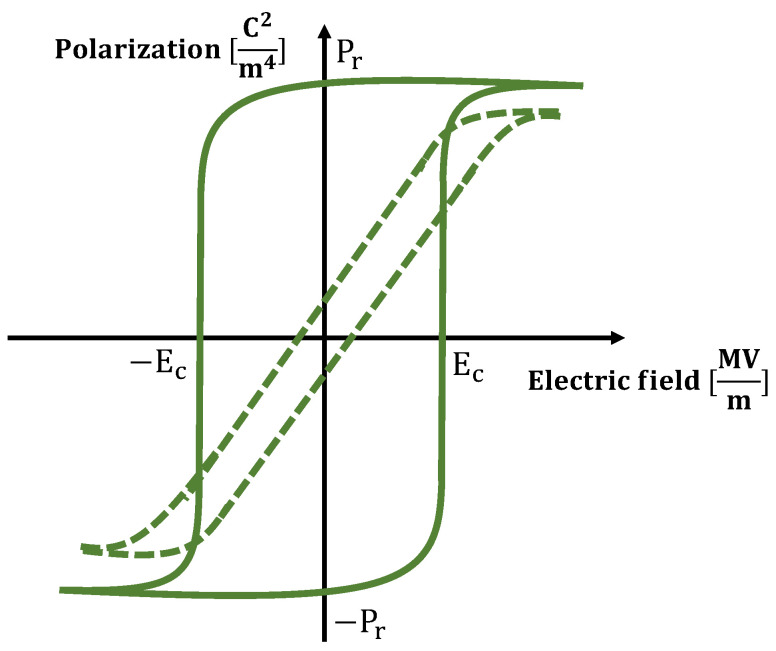
Electric field polarization hysteresis loops for a ferroelectric polymer (e.g., the PVDF, solid line) and for a relaxor ferroelectric polymer (e.g., the P(VDF-TrFE-CTFE), dashed line). P*_r_* and E*_c_* indicate the remnant polarization and the coercive field, respectively.

In the pursuit of designing a linear soft actuator with a high force-to-weight ratio and a stiffening behavior, this study analyzes the poly(vinylidene fluoride-trifluoroethylene-chlorotrifluoroethylene), i.e., the P(VDF-TrFE-CTFE), a relaxor ferroelectric polymer. The P(VDF-TrFE-CTFE) has been chosen because it shows a high electrostrictive strain, i.e., the ability to produce a large field-induced strain when exposed to an external electric field [2,3]. The P(VDF-TrFE-CTFE) is a terpolymer of the ferroelectric polymer polyvinylidene fluoride (PVDF) [4], in which the introduction of TrFE and CTFE facilitates the flipping of the dipoles [5] and the formation of trans-conformation, respectively [6]. As a result, the P(VDF-TrFE-CTFE) is a relaxor ferroelectric polymer, with low Curie temperature and high dielectric constant. Figure 1 sketches the electric field-polarization hysteresis loops for a ferroelectric polymer (e.g., the PVDF, solid line) and for a relaxor ferroelectric polymer (e.g., the P(VDF-TrFE-CTFE), dashed line) [7,8]. It can be noted that, for a relaxor ferroelectric polymer, such as the one considered in this study, it is possible to orient dipoles and, therefore, electrostrictive strain with lower electric fields, making it an interesting electro-active material for soft actuation.

This study investigates whether the electrostriction of the P(VDF-TrFE-CTFE) and, as a consequence, its actuation capabilities can be enhanced by organizing it in aligned nanofibers through the electrospinning technique [9]. The underpinning idea of organizing the P(VDF-TrFE-CTFE) in aligned nanofibers through electrospinning is to facilitate the mobility of the dipoles (enforced by the nanofibrous form) and to induce the high density of dipoles (enforced by the aligned configuration, which is realized by mechanically stretching the nanofibers during electrospinning) [10]. The effect of changes in the morphology of polymers, processed either in thin films or electrospun nanofibers mats, has been analyzed for several applications, such as fluorescent sensors [11], membranes with tuned mechanical properties [12], or sensors for the detection of explosives [13]. To analyze and measure the electrostrictive effect, an external electric field should be applied to the polymeric specimen. However, when electrodes are bonded to the surface of the material, the overall measured strain is given by the synergistic effects of the strain induced by the Maxwell stress, which describes the Coulomb interactions of the charges between the electrodes, and the electrostrictive strain. In a previous study [14], these synergistic effects have been exploited in the design of variable stiffness spring, in which P(VDF-TrFE-CTFE) mats of aligned nanofibers have been interleaved between electrodes and passive layers.

In general, the strain induced by the Maxwell stress is greater than the strain induced by electrostriction, especially when high electric fields are applied [15,16]. Therefore, to evaluate electrostriction, it is required to experimentally measure it separately from the Maxwell stress. Due to the experimental challenges in separating the two effects, mathematical models have been used in, e.g., poly(vinylidenefluoride)-based polymers [17] and ideal polar rubbers [18], or different approaches have been proposed to determine the electrostrictive coefficients [19], i.e., electrostrictive coefficients are derived by measuring the strain-polarization and the strain-electric field curves [20,21], or by using the relationship between dielectric permittivity versus the applied stress [22], the piezoelectric coefficients, dielectric permittivities, and spontaneous polarization [23], the lattice parameters [24], the dielectric permittivity under a DC-biased electric field [25].

In this paper, an experimental test set-up has been designed that allows measuring the electrostrictive effect disjointly from the Maxwell stress. Namely, the electrodes used to apply the electric field are in contact with the P(VDF-TrFE-CTFE) but not bonded to the specimens’ surfaces. The electro-mechanical characterization is performed on P(VDF-TrFE-CTFE) specimens in the form of both film and aligned electrospun nanofiber mat, and is aimed at highlighting how these forms influence the force-to-weight ratio and the stiffening behavior when the specimens are stimulated by an increasing external electric field. Specifically, an *uniaxial load test* is designed that keeps the displacement of the polymeric specimen constant while the electrostrictive effect, i.e., the contraction force caused by the external electric field, is recorded. This electro-mechanical analysis shows that both the P(VDF-TrFE-CTFE) film and the P(VDF-TrFE-CTFE) nanofiber mat are electrostrictive, and that the aligned nanofiber mat has a higher electrostrictive effect than the films. Afterwards, an *uniaxial tensile load test* is designed that varies the displacement of the polymeric specimen while the polymer’s forces, resulted from the reaction to the tensile test itself and from the contraction of the material when the external electric field is applied, are recorded. This electro-mechanical analysis shows that when the external electric field increases, both the P(VDF-TrFE-CTFE) film and the P(VDF-TrFE-CTFE) nanofiber mat exhibit a stiffening behavior, which is more evident for the aligned nanofibers mat than the film and is proportional to the weight of the material. For both tests, a reference dielectric material i.e., the perfluoroalkoxy alkane (PFA), has been used.

To summarize, the main contributions of this study are (i) to present an experimental set-up for the measurement of the electrostrictive effect of polymeric specimens disjointly from the Maxwell stress; (ii) to experimentally show that the P(VDF-TrFE-CTFE) is electrostrictive in both forms of film and aligned nanofibers mat; (iii) to demonstrate that the aligned nanofibers mat has a greater contraction capability than the film thanks to the nanostructuring; (iv) to measure the stiffening behavior of the film and the nanofibers mat when stimulated by an electric field and a mechanical load; and (v) to highlight that the P(VDF-TrFE-CTFE) has high potential to be used as electro-active polymer for soft actuators because of its high force-to-weight ratio and stiffening behavior, which are both further enhanced by organizing the polymer in aligned nanofibers.

The remainder of the paper is organized as follows. Section 2 presents the polymers used in this study, i.e., the P(VDF-TrFE-CTFE) film, the P(VDF-TrFE-CTFE) electrospun nanofiber mat, and the PFA film. Section 3 describes the experimental test set-up that has been used for the electro-mechanical analysis of the specimens, by means of the uniaxial load test and the uniaxial tensile load test. The results are reported and discussed in Section 4. Finally, concluding remarks are drawn in Section 5.

## 2. Materials

This section presents the polymeric materials and specimens analyzed in this study, namely, the Solvene T^®^ P(VDF-TrFE-CTFE) and the PFA, both provided by Solvay Specialty Polymers (Solvay S.p.A., Milano, Italy, www.solvay.com).

### 2.1. P(VDF-TrFE-CTFE)

In this study, P(VDF-TrFE-CTFE) was chosen because of its high electrostrictive strain, i.e., the ability to produce a large field-induced strain when exposed to an external electric field [2,3]. When exposed to an electric field, the field-induced strain *S* of the material is

(1) $$S=QP^{2}$$

where *Q* is the electrostrictive coefficient and $P=D-\epsilon_{o}E$ the electric polarization, in which *D* is the electric displacement field, $\epsilon_{o}$ the vacuum permittivity, and *E* the electric field.

The Solvene T^®^ P(VDF-TrFE-CTFE) used in this study has 63 mol% of VDF, 28 mol% of TrFE, and 9 mol% of CTFE. Moreover, it has a dielectric constant $\epsilon_{r}=45$ at 1 kHz and a Curie temperature of 16 °C.

#### 2.1.1. P(VDF-TrFE-CTFE) Films

The film of P(VDF-TrFE-CTFE) has been realized by melt extrusion [26] and it has been cut to obtain specimens whose dimensions are reported in Table 1.

#### 2.1.2. P(VDF-TrFE-CTFE) Electrospun Nanofibers Mats

The nanofibers of P(VDF-TrFE-CTFE) have been realized by electrospinning technique [27]. Specifically, for the fabrication, a solution of P(VDF-TrFE-CTFE) powder (30 wt%) and Acetone:DMF 55:45 (w/w) was processed by an electrospinning machine (Spinbow^TM^, Bologna, Italy, www.spinbow.it/en), equipped with four needles (length of 55 mm and internal diameter of 0.84 mm) that are connected to 5 mL syringes via PTFE tubings. The nanofibers have been collected on a rotating drum, covered with poly(ethylene)-coated paper. The parameters of the electrospinning process are summarized in Table 2.

Two P(VDF-TrFE-CTFE) electrospun nanofiber mats have been fabricated for the experimental tests, one with an average thickness of ∼80 μm and another one with an average thickness of ∼60 μm. The mats have been cut to obtain specimens NF and specimens NF2, whose dimensions are reported in Table 1.

#### 2.1.3. SEM Analysis

**Figure 2:**
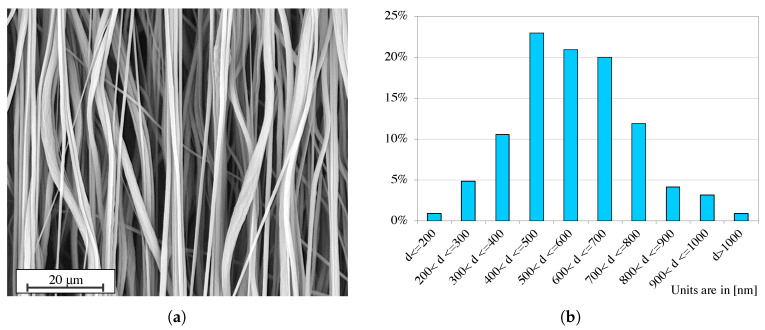
(**a**) SEM image of one specimen of the aligned nanofibers mat. (**b**) Histogram of the % distribution of the nanofibers’ diameters in three specimens.

The nanofiber mat was analyzed by scanning electron microscopy (SEM). A Phenom ProX Desktop SEM (Thermo Fisher Scientific, Waltham (MA), USA, www.thermofisher.com) was used to collect three images of three different specimens of the nanofiber mat. Figure 2a shows one image of a specimen of the aligned nanofiber mat. Figure 2b reports the histogram of the % distribution of the nanofibers’ diameters. The diameter analysis has been performed through the Phenom FiberMetric Software (Thermo Fisher Scientific, Waltham (MA), USA, www.thermofisher.com) considering about 300 nanofibers for each one of the three images. From the histogram, it can be noted that ∼23% of the nanofibers have a diameter between 400 and 500 nm.

### 2.2. PFA

In this study, the PFA has been chosen as the reference material because it is a dielectric material with low permittivity and, therefore, it is not electrostrictive. The PFA used in this study has a dielectric constant of $\epsilon_{r}=2.1$ (stable value from 1 kHz to 1 GHz).

#### PFA Films

The film of PFA has been realized by melt extrusion [26] and it has been cut to obtain specimens whose dimensions are reported in Table 1.

## 3. Method

This section describes the experimental test set-up for the electro-mechanical analysis of the specimens of the P(VDF-TrFE-CTFE) film, P(VDF-TrFE-CTFE) electrospun nanofibers mat, and PFA film.

### 3.1. Experimental Test Set-Up

An experimental test set-up has been designed to measure the electrostrictive effect of a polymeric specimen, i.e., the contraction force produced by the specimen when stimulated by an electric field, disjointly from the Maxwell stress. Specifically, the electrodes used to apply the electric field are in contact with the specimen, but not bonded to the specimen’s surface, and are located at a fixed position.

**Figure 3:**
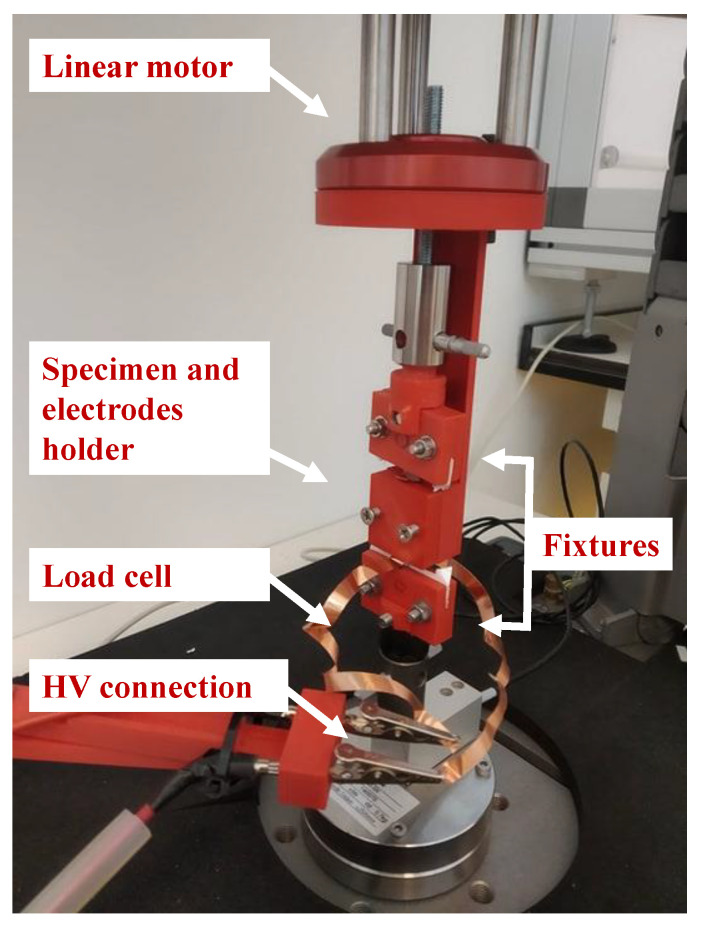
Specimen in the Instron test instrument. The fixtures and the support for the specimen (zoomed in the figure) are rigidly connected to the linear motor at the top. The contraction force of the specimen is registered by the Instron load cell placed at the bottom.

The set-up consists of the test instrument ElectroPuls E1000 (Instron™, Norwood (MA), USA, www.instron.us), equipped with an optical encoder and the Instron™ static load cell 2530-5N, which has a capacity of 5 N and a sensitivity of 1.6 mV/V to 2.4 mV/V at static rating. Figure 3 shows how the specimen is mounted in the Instron test instrument, i.e., the specimen is held by means of two fixtures, which have been 3D printed in ABS material. To apply the electric field to the specimen, a 3D printed electrodes holder has been realized that can internally host the electrodes (of dimensions 30×7×0.07 mm) and the specimen in a groove of about 0.2 mm depth. The electrodes are glued on the 3D printed holder and are in contact with the specimen but are at a fixed position, which allows for the contraction of the specimen with negligible friction. The presence of air between the electrodes and the specimen is limited, preventing dielectric breakdown at the values of the applied electric field.

**Figure 4:**
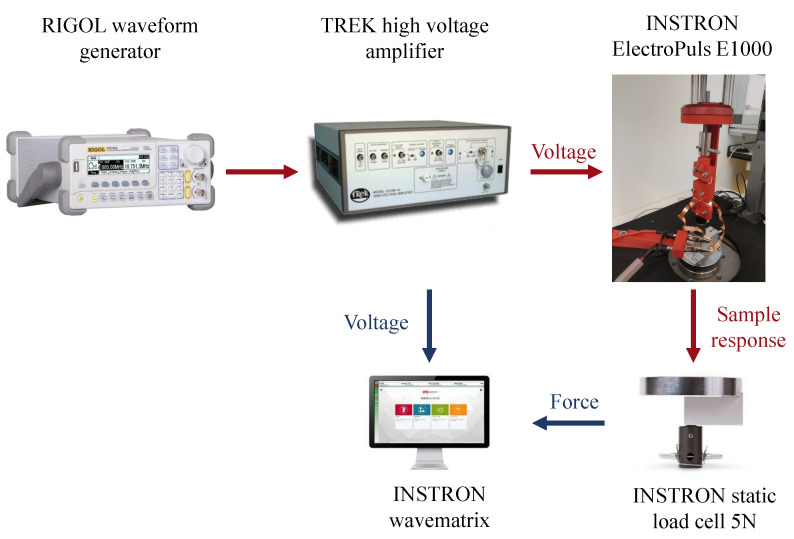
Overall experimental test set-up for the electro-mechanical characterization. The TREK 10/10B-HS high-voltage amplifier, operated through the RIGOL DG1022 waveform generator, stimulates the specimen. The contraction force of the specimen is measured by the Instron load cell and recorded by the Instron™ Wavematrix software.

A 10/10B-HS high-voltage amplifier (Trek Inc., Lockport (NY), USA, www.trekinc.com) is connected through crocodile plugs to the electrodes and is operated through the DG1022 waveform generator (RIGOL Technologies, Beaverton (OR), USA, www.rigolna.com). The Instron™ Wavematrix software records both the forces measured by the Instron load cell and the applied voltage. Figure 4 shows the overall experimental test set-up.

### 3.2. Experiment 1: Uniaxial Load Test

The *uniaxial load test* is designed to keep the displacement of the polymeric specimen constant while the forces, resulting from the contraction of the material when an external electric field is applied, are recorded.

**Figure 5:**
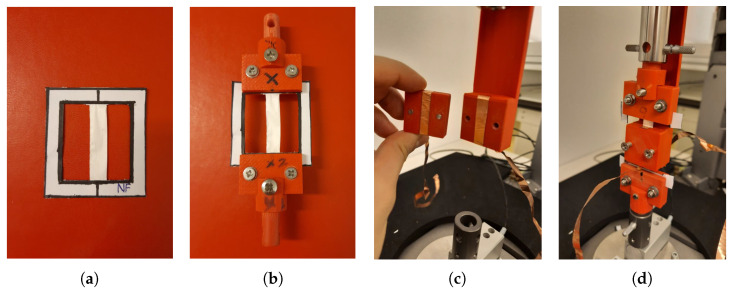
Placement steps of the specimen in the Instron test instrument. (**a**) The specimen anchored into the paper frame. (**b**) The paper frame is clamped. (**c**) The electrodes are glued into the internal groove of the 3D printed electrodes holder. (**d**) The clamped specimen is inserted into the groove of the 3D printed electrodes holder and inside the Instron test instrument. Finally, the paper frame is cut on the sides before the preloading.

The specimen is placed in the Instron test instrument by following the procedure proposed in [28,29], which avoids damaging the specimens. First, the specimen is anchored into a paper frame with a bi-adhesive tape to prevent slippage. Then, the paper frame is clamped in the Instron test instrument and, afterwards, the paper frame is cut on the sides, leaving the specimen properly placed in the test instrument, with a free length of 40 mm. Finally, the specimen is preloaded at 0.1 N. This preload value is chosen for all the materials, in order to exclude for this study the preload influence on the electrostriction effect. The complete procedure for the positioning of the specimen in the Instron test instrument is shown in Figure 5.

**Figure 6:**
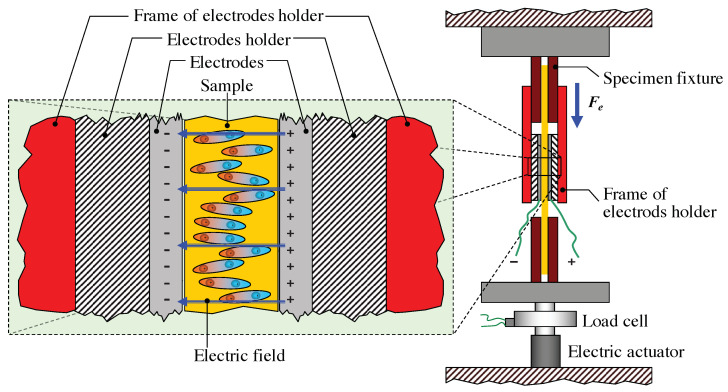
Uniaxial load test: the specimen (orange), inserted into the electrodes holder (dashed black and red), is held by two fixtures (dark red), which are kept at a fixed position. When an external electric field is applied, the specimen contracts and the load cell measures the contraction force $F_{e}$.

When the test starts, the specimen is stimulated by different electric fields and, as a consequence, it contracts perpendicularly to the field [30]. The uniaxial load test is sketched in Figure 6, where the specimen (orange), inserted into the electrodes holder (black red and red), is held by two fixtures (dark red) at a fixed position and the contraction force $F_{e}$, perpendicular to the electric field, is measured by the load cell.

We assume that the contraction force $F_{e}$, i.e., the electrostrictive effect, can be described by [31]

(2) $$F_{e}=K\left(E\right)S$$

where K(E) is the field-dependent stiffness of the polymeric specimen and *S* the electrostrictive strain, given by Equation (1). By substituting Equation (1) in Equation (2), it follows that

(3) $$F_{e}=K\left(E\right)QP^{2}$$

### 3.3. Experiment 2: Uniaxial Tensile Load Test

The *uniaxial tensile load test* is designed that varies the displacement of the polymeric specimen while the polymer’s forces, resulted from the reaction to the tensile test itself and from the contraction of the material when an external electric field is applied, are recorded.

The specimen is placed in the Instron test instrument, with the same procedure as in the uniaxial load test, and preloaded at 0.1 N. After preloading, the displacement of the specimen is changed as follows.

- The displacement between the fixtures is kept constant for 10 s and the desired electric field is applied.
- The displacement between the fixtures is changed by applying an ascending ramp of 0.6 mm with a rate of 0.01 mm/s.
- The displacement between the fixtures is changed by applying a descending ramp of 0.6 mm with a rate of 0.01 mm/s.

The specimen is stimulated by different electric fields and, as a consequence, it contracts perpendicularly to the field [30]. In this case, the load cell measures the contributions of the tensile reaction force $F_{m}$ of the material, due to the changes in the displacement between the fixtures, and the contraction force $F_{e}$, perpendicular to the electric field. Therefore, the total force $F_{t}$ measured by the load cell is

(4) $$F_{t}=F_{m}+F_{e}$$

**Figure 7:**
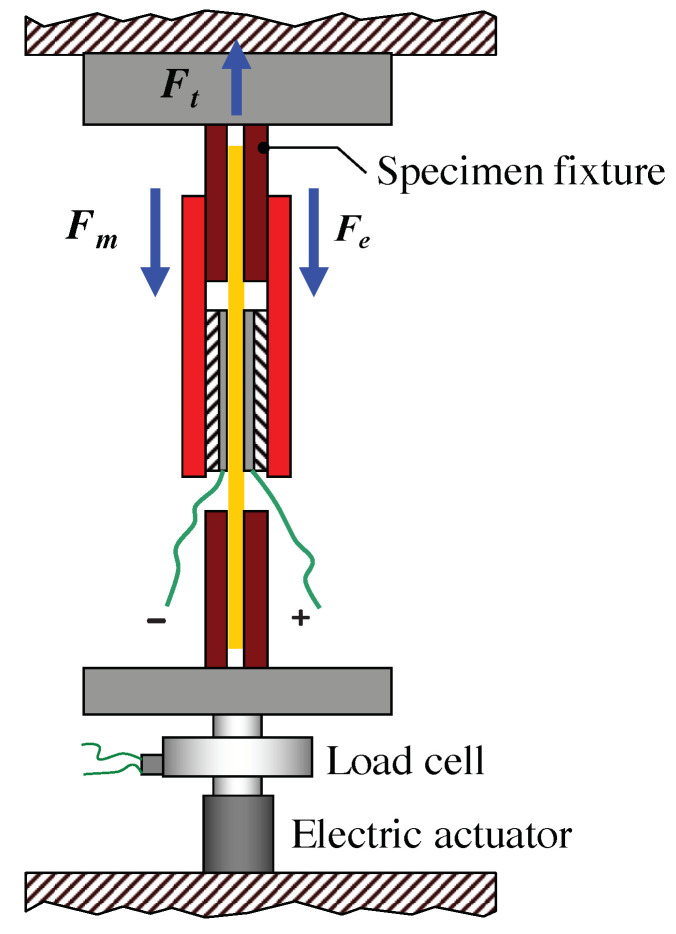
Uniaxial tensile load test: the specimen (orange), inserted into the electrodes holder (dashed black and red), is held by two fixtures (dark red). During the test, the upper fixture is moved upwards. When an external electric field is applied, the specimen contracts and the load cell measures the total force $F_{t}$, given by the contributions of both the tensile reaction force $F_{m}$ of the material and the contraction force $F_{e}$, as described by Equation (4).

The uniaxial tensile load test is sketched in Figure 7, where the specimen (orange), inserted into the electrodes holder (dashed black and red), is held by two fixtures (dark red), whose position changes during the test, and the total force $F_{t}$ is measured by the load cell.

It is important to note that the displacement range between the fixtures is chosen to maintain the polymeric specimens in the elastic phase of the materials, and so to repeat the test sequence, at least, three times on the same specimen. Moreover, before acquiring the data, a complete cycle without any applied electric field is performed to stabilize the mechanical behavior of the specimen. After this first cycle, the uniaxial tensile load test starts for nine different electric fields from 0 MV/m to 15 MV/m.

## 4. Results and Discussion

This section reports and discusses the results of the uniaxial load tests and of the uniaxial tensile load tests on the materials considered in this study.

### 4.1. Results Experiment 1: Uniaxial Load Test

**Figure 8:**
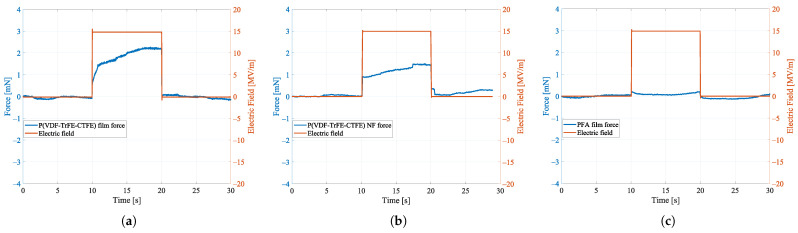
Contraction forces in the uniaxial load test for (**a**) the specimen of P(VDF-TrFE-CTFE) film, (**b**) the specimen of P(VDF-TrFE-CTFE) nanofibers (NF) mat, and (**c**) the specimen of PFA film.

Figure 8 reports the contraction forces $F_{e}$, which have been measured in the uniaxial load tests on the specimen of the P(VDF-TrFE-CTFE) film, the specimen of P(VDF-TrFE-CTFE) nanofibers (NF) mat, and the specimen of PFA film when an electric field of 15 MV/m is applied. This specific value of applied electric field is sufficiently high to demonstrate the electrostrictive properties of the P(VDF-TrFE-CTFE)-based materials [17], while avoiding the risk of dielectric breakdown especially in the nanofibers mat, which is characterized by low density. In the figure, it can be noted that, after a transient of ∼10 s from the application of the electric field, the contraction forces of the P(VDF-TrFE-CTFE)-based specimens reach a steady state, whereas, as expected, the PFA film specimen does not show any contraction force. This electro-mechanical analysis shows that both the P(VDF-TrFE-CTFE) film and the nanofibers mat are electrostrictive. Specifically, when stimulated by an electric field of 15 MV/m, the film with a weight of 57.7 mg can exert a contraction force of ∼2.2 mN, while the nanofibers mat with a weight of 23.2 mg can exert a contraction force of ∼1.5 mN.

**Figure 9:**
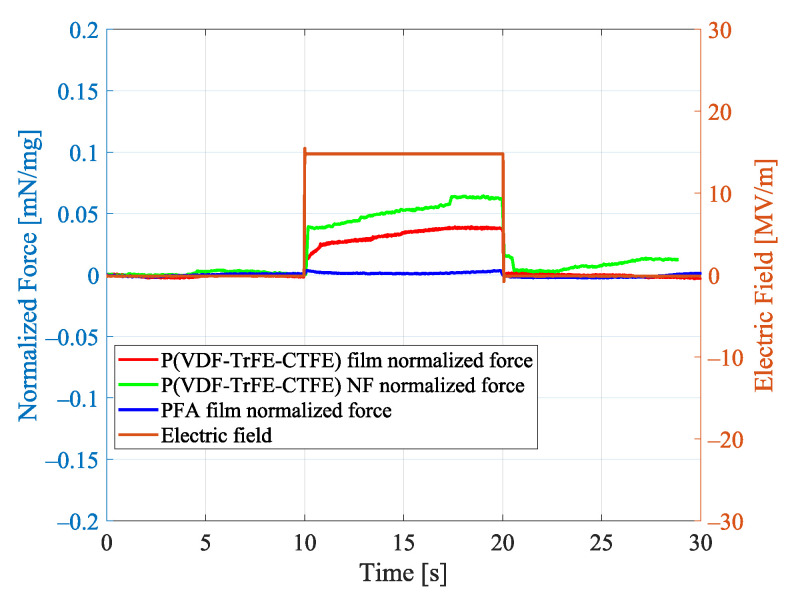
Normalized contraction forces in the uniaxial load test for the P(VDF-TrFE-CTFE) film, the P(VDF-TrFE-CTFE) NF nanofibers mat, and the PFA film.

Figure 9 reports the same results normalized to the quantity of polymeric material, i.e., divided by the weight of the specimen. It can be noted that, when normalized to the weight of the material, the contraction force of the P(VDF-TrFE-CTFE) nanofibers mat is higher than the contraction force of the P(VDF-TrFE-CTFE) film. Specifically, when stimulated by an electric field of 15 MV/m, the film has a force-to-weight ratio of ∼0.04 mN/mg, while the nanofiber mat has a force-to-weight ratio of ∼0.068 mN/mg. Consequently, the nanofiber mat exhibits a force-to-weight ratio ∼65% higher than the film. This electro-mechanical analysis shows that the form of aligned nanofibers mat, produced by the electrospinning technique, enhances the electrostrictive property of the polymer.

**Figure 10:**
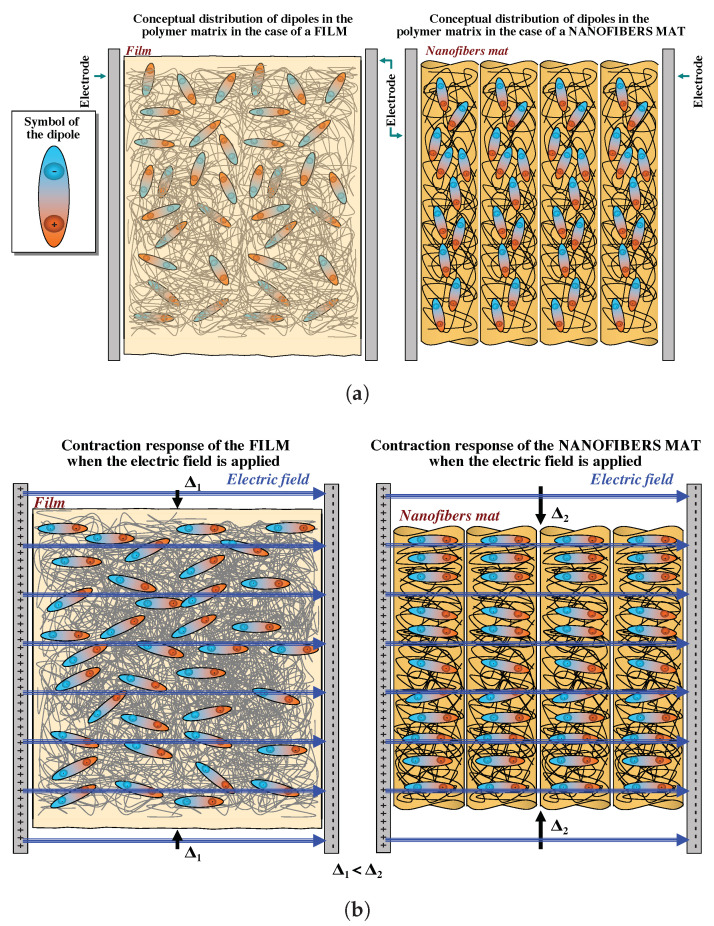
Schematic representation of a film and a nanofibers mat. (**a**) Randomly oriented dipoles in the film and in the nanofibers mat without any applied electric field. (**b**) When an electric field is applied, the dipoles are oriented according to the electric field, and therefore both specimens contract. The nanofibers shows a larger contraction $\Delta_{2}$ when compared to the contraction $\Delta_{1}<\Delta_{2}$ of the film.

In general, the electrostriction of a dielectric material can be increased by increasing its dielectric permittivity [32]. Electrospinning is a process that enhances the dielectric properties of the material for the following main reasons: (i) the mechanical stretching enforced by the electrospinning process enables for the alignment of the polymeric chains in the amorphous phase; (ii) the nanofibers have dipoles with a self-induced orientation; (iii) the electrical poling also enforced by the electrospinning process enables the spontaneous dipolar orientation [33] inside the nanofibers; and (iv) the nanofibers have a larger surface area to volume ratio than a bulk, which also increases the dielectric constant [34]. Furthermore, nanofibers allow for a better alignment of the dipoles because of the smaller amount of material that has to be reorganized around each dipole. In the film, the orientation of the dipoles is statistically influenced by a greater amount of material than in the nanofibers mat. In Figure 10, a schematic representation of this phenomenon is given. Specifically, Figure 10a shows the film (left) and the nanofiber mat (right) placed between two electrodes without any applied electric field. The dipoles therein are randomly oriented. When an electric field is applied, as depicted in Figure 10b, the dipoles are oriented according to the electric field. However, the nanofibers mat shows a larger contraction $\Delta_{2}$ when compared to the contraction $\Delta_{1}<\Delta_{2}$ of the film, thanks to the smaller amount of material.

**Figure 11:**
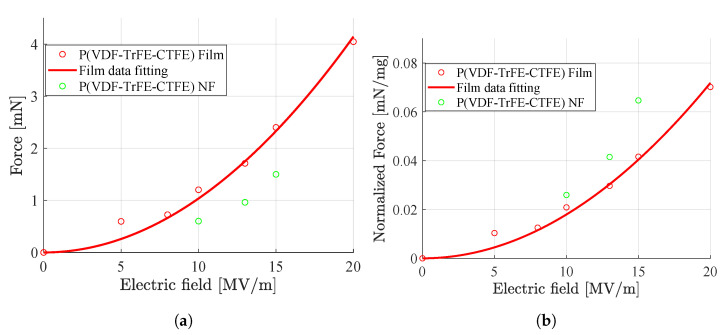
Maximum contraction forces registered at steady state (i.e., after ∼10 s) in the uniaxial load test for the P(VDF-TrFE-CTFE) film and the P(VDF-TrFE-CTFE) nanofiber (NF) mat for different applied electric fields. (**a**) Maximum contraction forces. (**b**) Maximum normalized contraction forces.

Finally, in the uniaxial load test, for both the film and NF nanofibers mat, it was possible to calculate the maximum contraction forces $F_{e}$ at steady state (i.e., after ∼10 s from the application of the electric field) for different electric fields. The same values are also normalized to the quantity of polymeric material, i.e., divided by the weight of the specimen, showing an higher force-to-weight ratio for the nanofiber mat for all the analyzed electric field values. The results are reported in Figure 11, where it can be noted that, as expected due to electrostriction, there is a quadratic relation between the contraction forces and the applied electric fields. From the Figure, it can be noted that the quadratic fitting has been done only for the film. For values of applied electric fields lower than 10 MV/m, it was not possible to detect a clear contraction in the nanofibers mat, supposedly for the higher sensitivity of the nanofibers mat to the effect of friction in the test setup. Moreover, for the nanofibers mats, it was not possible to apply an electric field higher than 15 MV/m because, due to a high specific surface area and high porosity of the nanofibers [35], the mat has a lower dielectric strength compared to the film. The integration of the electrospun nanofibers mat inside a silicon matrix [36] can enhance the dielectric strength of the mat. However, this configuration would increase the overall stiffness of the resulting specimen and, most likely, would reduce its contraction capability.

#### Aligned Nanofibers Mat: Repeatability

**Figure 12:**
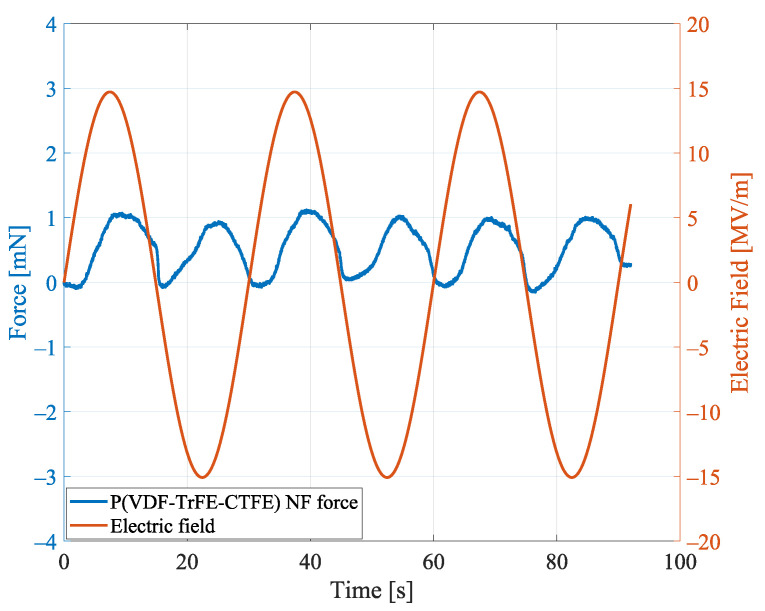
Contraction forces in the uniaxial load test for the P(VDF-TrFE-CTFE) nanofiber (NF) mat for a sinusoidal applied electric field.

To show repeatability in the uniaxial load test for the specimen of aligned nanofiber mat (NF), Figure 12 reports the contraction forces when a sinusoidal electric field, with an amplitude of 15 MV/m and a period of 30 s, is applied.

In the figure, it can be noted that the contraction forces of the specimen follow the applied sinusoidal input. Moreover, the forces are always positive, i.e., the specimen contracts, independently of the sign of the applied electric field. This behavior is due to the electrostrictive effect and, specifically, to the quadratic relationship between the contraction force and the electric polarization, as described by Equation (3). The average values of the six peaks forces is 1.101 mN and the standard error is below 0.035 mN, which shows repeatability of the exerted contraction forces of the specimen when the same electric field is repeatedly applied over time.

The electrostrictive effect and, therefore, the contraction forces for the aligned nanofibers mat are strictly related to the density of the nanofibers and to the alignment of the fibers. These properties are related to the fabrication process and specimen-specific.

### 4.2. Results Experiment 2: Uniaxial Tensile Load Test

**Figure 13:**
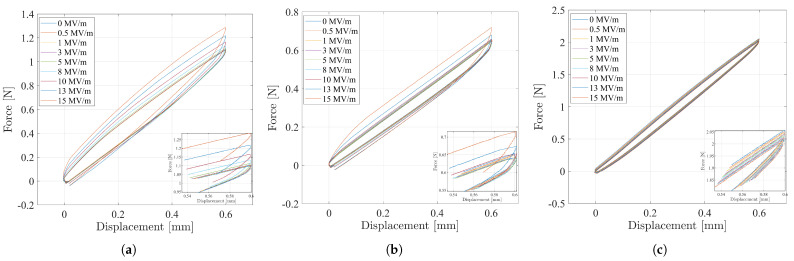
Force–displacement plots in the uniaxial tensile load test for (**a**) the specimen of P(VDF-TrFE-CTFE) film, (**b**) the specimen of the P(VDF-TrFE-CTFE) nanofiber (NF) mat, and (**c**) the specimen of the PFA film.

Figure 13 reports the total forces $F_{t}$ against the displacements, which have been measured in the uniaxial tensile load tests on the specimen of the P(VDF-TrFE-CTFE) film, the specimen of the P(VDF-TrFE-CTFE) nanofibers (NF) mat, and the specimen of PFA film, when different electric fields have been applied. It can be noted that, at a given displacement, the P(VDF-TrFE-CTFE)-based specimens show an increasing total force $F_{t}$ for increasing electric field. Specifically, the slopes, given by the interpolation of the force–displacement plot during the complete cycle of contraction and relaxation for each applied electric field, describe the stiffnesses σ of the specimens at different electric fields.

While the stiffness of the PFA is constant at 3.4 N/mm, the stiffnesses of the P(VDF-TrFE-CTFE)-based specimens changes, as reported in Table 3, where the reported stiffness is the average stiffness calculated on (at least) three uniaxial tensile load tests on each specimen. From the table, it can be noted that for the P(VDF-TrFE-CTFE) nanofiber (NF) mat, the electrostrictive effect on the stiffness is evident when the applied field exceeds 5 MV/m, i.e., below 5 MV/m the stiffness can be considered constant, as the recorded values are within the standard error.

**Figure 14:**
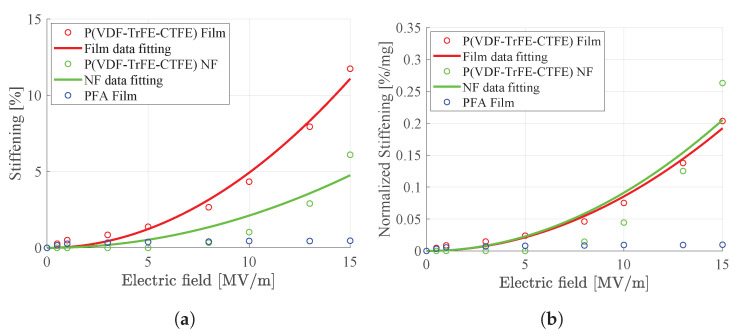
Uniaxial tensile load test for the P(VDF-TrFE-CTFE) film, the P(VDF-TrFE-CTFE) NF nanofiber mat, and the PFA film. (**a**) Stiffening plot. (**b**) Normalized stiffening plot.

Figure 14 shows, for different electric fields, the percentage increase in the stiffness of the specimens of the P(VDF-TrFE-CTFE) film, P(VDF-TrFE-CTFE) nanofibers (NF) mat, and PFA film. In particular, Figure 14a reports the stiffening of the specimens, calculated as the percentage increase of the stiffness for different electric fields with respect to the stiffness of the specimens without any applied field. Figure 14b reports the stiffening of the different specimens, normalized to the weight of the material. It can be noted that, when the external electric field increases, the P(VDF-TrFE-CTFE)-based specimens show a stiffening behavior, whereas, as expected, the PFA film does not show any stiffening behavior if small stiffness variations are excluded, as all dielectrics exhibit an electrostrictive effect [37].

From the figure, it can also be noted that, for the P(VDF-TrFE-CTFE)-based specimens, there is a quadratic dependency of the stiffness increment with respect to the applied electric fields. This quadratic dependency is due to electrostriction (see Equation (1)) and can be described by

(5) $$\sigma =pE^{2}$$

where σ is the material stiffening, *E* the electric field, and *p* a coefficient that has to be fitted by the experimental data.

This electro-mechanical analysis confirms that the specimens of both the P(VDF-TrFE-CTFE) film and P(VDF-TrFE-CTFE) nanofibers mat are electrostrictive. Moreover, it shows that, when the external electric field increases, both the film and the nanofibers mat exhibit a stiffening behavior, which is more evident for the nanofibers mat than for the film, when normalized to the weight of the material.

**Figure 15:**
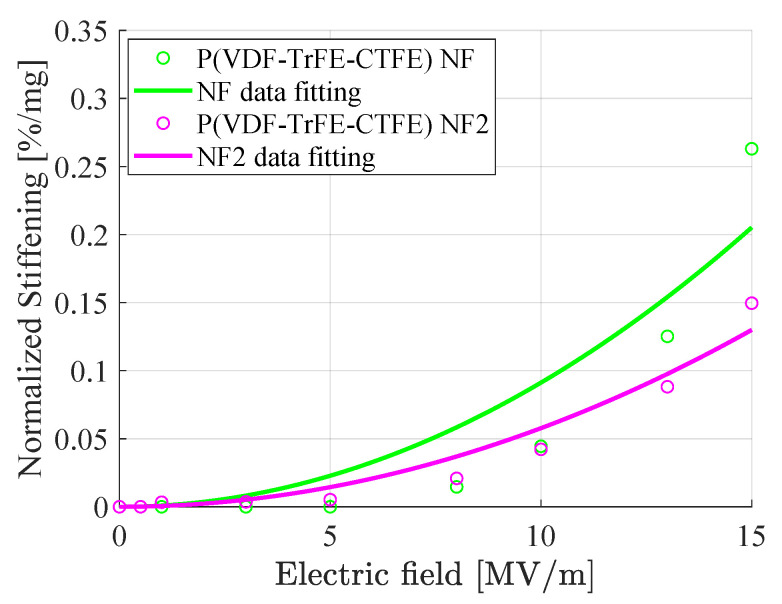
Normalized stiffening plot in the uniaxial tensile load test for two P(VDF-TrFE-CTFE) nanofibers (NF and NF2) mats. NF weights 0.0232 g, while NF2 0.0166 g (see Table 1).

Figure 15 shows, for different electric fields, the normalized percentage increase of the stiffness of two specimens of the P(VDF-TrFE-CTFE) nanofibers mats with different thicknesses, i.e., the specimen NF and the specimen NF2, with the dimensions as specified in Table 1. It can be noted that the stiffening behavior of the thicker specimen is greater than the one of the thinner specimen. This electro-mechanical analysis shows that the stiffening is proportional to the weight and, therefore, the quantity of the electro-active material.

**Figure 16:**
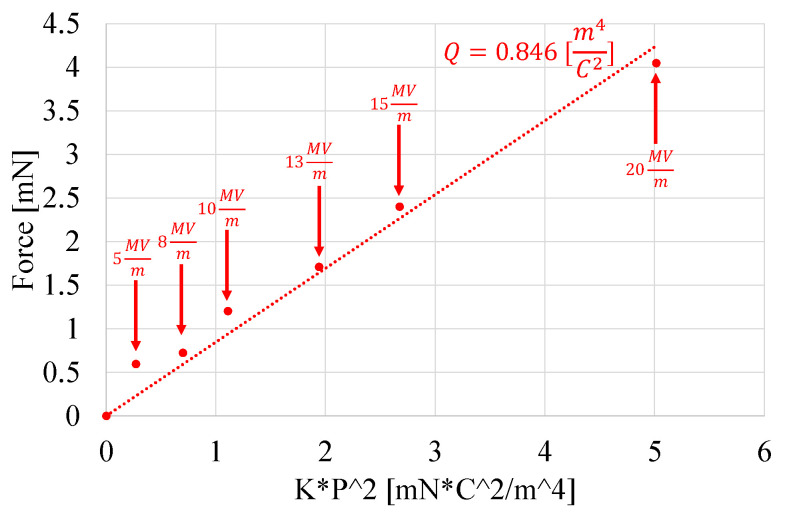
Linear fitting of electrostrictive coefficient *Q* for the P(VDF-TrFE-CTFE) film (see Equation (3)).

Figure 16 derives the value of electrostrictive coefficient *Q* for the P(VDF-TrFE-CTFE) film by using Equation (3). In particular, the maximum contraction forces $F_{e}$ (measured at steady state, i.e., after ∼10 s from the application of the electric field, in the uniaxial load test for the P(VDF-TrFE-CTFE) film against different electric fields and reported in Figure 11a) have been plotted against the field-dependent stiffness K(E) (experimentally derived and reported in Figure 14a) multiplied by the square of the polarization *P*. It can be noted that the electrostrictive coefficient Q is experimentally calculated by linear fitting and has a value of 0.846 m^4^/C^2^ [22,38].

The electrostrictive coefficient *Q* was derived only for the film due to the limitations of the nanofibers mat, as discussed in Section 4.1. Nevertheless, the electrostrictive coefficient *Q* of the nanofibers mats would be specimen-specific and, therefore, not generalizable as a property of the material because of the variation in the density of the nanofibers in different specimens due to the fabrication process.

### 4.3. Future Outlook: P(VDF-TrFE-CTFE)-Based Soft Actuators

In this section, the results obtained with the proposed electromechanical characterization are summarized and discussed with respect to the recent literature. Moreover, in the pursuit of designing a linear soft actuator, a future outlook for the studied P(VDF-TrFE-CTFE) nanofiber mat is given.

#### 4.3.1. Force-to-Weight Ratio

In recent literature, it has been shown that soft actuators, realized by aligned cellulose nanofibers, generate a twofold greater force-to-weight ratio when compared to a random nanofibers structure of the same material [39]. Moreover, electrospinning has been used to fabricate soft actuators with enhanced actuation properties thanks to the specific orientation of thermoresponsive hydrogel fibers [40].

This study shows that by organizing the P(VDF-TrFE-CTFE) in an aligned nanofiber mat by means of an electrospinning technique, it is possible to increase its force-to-weight ratio by ∼65% with respect to a film. It is, therefore, possible to conclude that electrostrictive polymers, organized in aligned fibrous structures, can have an important role for the force generation in soft actuators.

#### 4.3.2. Stiffening

One of the core challenges in soft robotics research is the variability and controllability of the robot’s overall compliance [41]. Several materials and methods have been employed to achieve a variable stiffness in soft actuators, such as combining dielectric elastomers with low melting point alloys [42], using shape memory polymer composite tubes [43], fabricating suitable contact surfaces for conducting polymer actuators [44].

This study shows that the P(VDF-TrFE-CTFE), with electrodes in contact with the specimen but located at a fixed position, exhibits a stiffening behavior when stimulated by increasing electric fields, which is more evident for the aligned nanofibers mat than for the film. Specifically, at an applied electric field of 15 MV/m, the P(VDF-TrFE-CTFE) shows a stiffening of ∼11.7% for the film and ∼6% for the nanofiber (NF) mat. During the experiments the electrodes are at a fixed position and, therefore, the obtained stiffening is not caused by the Maxwell stress due to the electrodes [45], but by a direct dependency of the material’s stiffness with the applied electric field.

The study also shows that the weight of the material influences the stiffening. In fact, it is possible to notice that for an applied field of 15 MV/m a stiffening of ∼0.204%/mg for the film, ∼0.263%/mg for the nanofibers mat NF, and ∼0.150%/mg for the nanofibers mat NF2.

#### 4.3.3. Scalability

Recent literature has focused on investigating parallel fibrous topologies, e.g., bundles of (nano)fibers, to realize linear soft actuators [46,47], whose contraction capabilities are enhanced by the organized structure [9].

Electrospinning has been used to fabricate sub-micron silk fibroin fibers with high homogeneity and large surface area-to-volume ratios in [47]. The highly organized parallel actuator’s structure, given by the bundle, enhances its mechanical and electrical properties, and generates peak stresses of ∼70 kPa (for a diameter that ranges from 200 to 500 μm).

The research on parallel fibrous topologies is still ongoing, but bundling the nanofibers mat can be a promising method to further increase the force-to-weight ratio of the bundle-based soft actuator.

## 5. Conclusions

This study analyzed the electrostrictive property of the P(VDF-TrFE-CTFE) polymer in the form of both film and aligned electrospun nanofibers mat.

The electrostrictive effect of the polymer is measured disjointly from the Maxwell stress thanks to a test set-up that keeps the electrodes (used to apply the electric filed) in contact with the specimens but located at a fixed position, i.e., not bonded to the specimen. Two different experiments have been conducted. The uniaxial load test is designed that keeps the displacement of the polymeric specimen constant while the forces, resulted from the contraction of the material when an external electric field is applied, are recorded. This electro-mechanical test shows that both the P(VDF-TrFE- CTFE) film and the aligned nanofibers mat are electrostrictive, and that the mat has a higher electrostrictive effect than the film. The uniaxial tensile load test is designed that varies the displacement of the polymeric specimen while the polymer’s forces, resulted from the reaction to the tensile test itself and from the contraction of the material when an external electric field is applied, are recorded. This electro-mechanical test shows that, when the external electric field increases, both the P(VDF-TrFE-CTFE) film and the aligned nanofibers mat exhibit a stiffening behavior, which is more evident for the mat than for the film.

From this study, it is possible to conclude that the P(VDF-TrFE-CTFE), thanks to its high force-to-weight ratio and the stiffening behavior, further enhanced in form of an aligned electrospun nanofibers mat, has high potential to be used as electro-active polymer for soft actuators in biomedical and biorobotic applications. The study of P(VDF- TrFE-CTFE)-based soft actuators is, nevertheless, left for future work.

## Figures and Tables

**Table 1:** Dimensions (Length l, Breadth b, Thickness t, Weight) of the specimens of P(VDF-TrFE-CTFE) film, P(VDF-TrFE-CTFE) electrospun nanofibers (NF and NF2) mats, and PFA film.

Material	l [mm]	b [mm]	t [μm]	w [mg]
P(VDF-TrFE-CTFE) film	55	9	∼60	57.7
P(VDF-TrFE-CTFE) NF	55	9	∼80	23.2
P(VDF-TrFE-CTFE) NF2	55	9	∼60	16.6
PFA film	55	9	∼55	48.1

**Table 2:** Electrospinning parameters used for the production of the P(VDF-TrFE-CTFE) nanofiber mats.

Flow rate [mL/h]	0.8
Electric potential [kV]	26
Drum rotation speed [rpm]	2430
Distance needles-drum [cm]	14
Relative humidity [%]	52
Temperature [°C]	20

**Table 3:** Stiffness of the specimens of the P(VDF-TrFE-CTFE) film and P(VDF-TrFE-CTFE) nanofiber (NF) mat. The reported data are the average values ± standard error.

Electric Field [MV/m]	Film Stiffness [N/mm]	Mat (NF) Stiffness [N/mm]
0	1.752±0.003	1.030±0.002
0.5	1.756±0.003	1.029±0.002
1	1.761±0.004	1.029±0.001
3	1.767±0.004	1.029±0.001
5	1.776±0.004	1.029±0.001
8	1.798±0.004	1.033±0.001
10	1.828±0.006	1.040±0.002
13	1.891±0.018	1.060±0.004
15	1.957±0.023	1.093±0.008

## Data Availability

The data presented in this study are available on request from the corresponding author.
